# Clinical study on modified Shenzhe Zhenqi Decoction combined with ambroxol hydrochloride in the treatment of severe pneumonia

**DOI:** 10.3389/fphys.2025.1601761

**Published:** 2025-07-16

**Authors:** Binyu Xing, Cunyi Shen, Ting Lin, Xiaoning Li, Wenjun Tan

**Affiliations:** Department of Surgery Intensive Care Unit, The First Affiliated Hospital of Xi’an Jiaotong University, Xi’an, Shaanxi, China

**Keywords:** severe pneumonia, Shenzhe Zhenqi Decoction, ambroxol hydrochloride, blood gas indices, lung function, inflammatory factors

## Abstract

**Objective:**

This study aimed to evaluate the clinical efficacy of modified Shenzhe Zhenqi Decoction combined with ambroxol hydrochloride in treating severe pneumonia.

**Methods:**

This was a single-center, prospective, double-blind, randomized controlled trial. A total of 138 patients with severe pneumonia were randomly divided into two groups. The control group (n = 69) received ambroxol hydrochloride, while the observation group (n = 69) received modified Shenzhe Zhenqi Decoction combined with ambroxol hydrochloride. Clinical efficacy, Traditional Chinese Medicine symptom scores (fever, cough, expectoration, dyspnea), arterial blood gas indicators [oxygen saturation (SaO_2_), partial pressure of oxygen (PaO_2_), partial pressure of carbon dioxide (PaCO_2_)], lung function [forced expiratory volume in one second (FEV1), forced vital capacity (FVC), peak expiratory flow (PEF)], and inflammatory markers [interleukin-6 (IL-6), tumor necrosis factor-α (TNF-α), C-reactive protein (CRP)] were evaluated. Adverse events were recorded for both groups.

**Results:**

The overall efficacy rates in the combined and control groups were 98.55% and 89.86% (*P* < 0.05). After treatment, in both groups, symptom scores for fever, cough, expectoration, and dyspnea were reduced, with the combined group showing more significant improvement; SaO_2_ and PaO_2_ levels increased, and PaCO_2_ decreased, with the combined group achieving greater improvement; FEV1, FVC, and PEF values increased, with higher values observed in the combined group; serum IL-6, TNF-α, and CRP levels decreased, with the combined group showing more substantial reductions (all *P* < 0.05). The incidence of adverse events was 4.35% in the combined group and 2.90% in the control group (*P* > 0.05).

**Conclusion:**

Modified Shenzhe Zhenqi Decoction combined with ambroxol hydrochloride demonstrated ideal clinical efficacy in treating severe pneumonia, alleviating symptoms and pulmonary inflammation and improving arterial blood gas indicators and lung function.

## Introduction

Pneumonia is a common and severe infection that can be triggered by a variety of pathogens, such as bacteria, viruses, fungi, or parasites (Shen et al., 2023). It is among the most frequent infections observed in elderly patients. For clinical purposes, pneumonias can be categorized based on where they are acquired: community-acquired, nursing home-acquired, or hospital-acquired pneumonias (Lanks et al., 2019). Community-acquired pneumonia is a prominent cause of death and illness worldwide, with significant clinical implications (Eshwara et al., 2020). Pneumonia affects individuals who are vulnerable, typically as a result of chronic factors (such as advanced age, smoking, diabetes, and numerous others) or acute occurrences (including intoxication, sudden spikes in air pollution, trauma, and so forth) (Mizgerd, 2017). Common signs and symptoms presented include rapid breathing, coughing, fever, and lack of appetite (Smith et al., 2021). When pneumonia is severe, it causes damage to the lungs.

The specific treatment for pneumonia depends on the type of pathogenic bacteria, the severity of the pneumonia, etc. Commonly used medications include cough suppressants, antipyretic and analgesic medications, and antibiotics. Ambroxol hydrochloride is a medication that exhibits multiple effects in the respiratory tract, including anti-inflammatory, antioxidant, antibacterial, antiviral, and immunomodulatory properties (Shen et al., 2023). It has been shown to be both effective and safe in treating a variety of respiratory diseases in both children and adults, such as bronchitis, cystic fibrosis, pneumonia, and tuberculosis (Shen et al., 2023). Administering ambroxol hydrochloride intravenously is a frequently used method to treat bronchopneumonia in adults (Liu et al., 2020). One study has found that nebulized inhalation of budesonide combined with ambroxol hydrochloride greatly improves C-reactive protein and procalcitonin levels in patients (Zhang et al., 2024). Additionally, research has disclosed that ambroxol hydrochloride is effective in dissolving sputum (Lee et al., 2019). Moreover, a different study has demonstrated that combining high-dose ambroxol hydrochloride with fiberoptic bronchoscopy helps improve blood gas indicators and lowers mortality rates in elderly patients with severe pneumonia (Tang et al., 2022).

Shenzhe Zhenqi Decoction is a traditional Chinese medicine formula with the effects of strengthening the body’s defenses, eliminating pathogenic factors, regulating Qi, and relieving adverse flow. Its main ingredients include ginseng, hematite, dragon bone, and oyster shell, and it is primarily used to treat symptoms such as cough and wheezing caused by Qi deficiency and blood stasis. Ginseng has been found to strengthen immunity against influenza in clinical trials and animal studies, and it also offers protection against pneumococcal pneumonia in animal experiments (Lee and Rhee, 2021). As the main components of medicine, ginseng and ocher also play a crucial role in respiratory diseases. Ochre is widely used in the treatment of bacterial infections due to its antioxidant properties and antimicrobial properties (Ali et al., 2022). Ginseng has anti-inflammatory, neuroendocrine modulating, immune system stimulating, and other effects (Zhao et al., 2024). Herein, we aimed to unravel the clinical efficacy of modified Shenzhe Zhenqi Decoction combined with ambroxol hydrochloride in patients with severe pneumonia so as to provide additional evidence for the comprehensive treatment of severe pneumonia.

## Materials and methods

### Ethics statement

Ethical approval has been obtained from the ethics committee of The First Affiliated Hospital of Xi’an Jiaotong University, and all enrolled cases have signed informed consent forms.

### Study design

This study was designed as a single-center, prospective, double-blind, randomized controlled trial.

### Basic information

A total of 138 patients with severe pneumonia admitted to The First Affiliated Hospital of Xi’an Jiaotong University from April 2022 to May 2023 were included. No statistically significant differences were found in baseline information between the two groups (*P* > 0.05), indicating comparability (Table 1).

**TABLE 1:** Comparison of basic information between the two groups.

Indicator	Combined group (n = 69)	Control group (n = 69)	*t/Z/χ* ^2^	*P*
Age (years)	52.72 ± 8.40	54.01 ± 7.20	−0.968	0.335
Duration of disease (d)	5.00 (4.00, 7.00)	5.00 (3.00, 6.50)	−0.806	0.420
Male/female (n)	47/22	43/26	0.511	0.475
Body mass index (kg/m^2^)	22.30 ± 1.28	22.77 ± 1.76	1.799	0.074
Hypertension [n (%)]	25 (36.23)	28 (40.58)	0.276	0.600
Diabetes [n (%)]	8 (11.59)	9 (13.04)	0.067	0.796
Hyperlipidaemia [n (%)]	24 (34.78)	21 (30.43)	0.297	0.586
Smoking [n (%)]	28 (40.58)	23 (33.33)	0.778	0.378
Alcohol drinking	20 (28.99)	15 (21.74)	0.957	0.328
Acute physiology and chronic health evaluation II score	19.00 (17.00,22.00)	21.00 (16.00,23.50)	−1.560	0.119

### Inclusion criteria

(1) Patients presenting symptoms such as cough, expectoration, fever, nasal congestion, runny nose, and dyspnea, with examination results confirming a diagnosis of severe pneumonia (Metlay et al., 2019); (2) Referring to the *Standards for Diagnosis and Therapeutic Effect of Traditional Chinese Medicine Diseases and Syndromes (ZY/T001.1-94)*, patients who exhibited symptoms of deficiency in both Yin and Yang, labored breathing, and signs of imminent collapse, meeting the indications for Shenqi Zhenqi Decoction therapy; (3) Patients who have been confirmed before enrollment to be free from immune system diseases; (4) Patients with complete clinical data; (5) Adults.

### Exclusion criteria

(1) Patients with concurrent pulmonary tuberculosis, severe pneumothorax, hemothorax, or malignant lung tumors; (2) Patients who suffered from severe cardiac, hepatic, renal, or hematological diseases or other malignancies; (3) Patients with severe mental disorders; (4) Patients with a history of allergy to any of the study drugs or related drug classes; (5) Patients unable to undergo pulmonary function tests; (6) Patients participating in other clinical trials; (7) Patients who did not adhere to the prescribed medication regimen, thus introducing confounding factors; (8) Patients unable to complete the treatment course due to uncontrollable external factors.

### Sample size calculation

The sample sizes of the combined group and the control group were allocated at a ratio of 1:1. The calculation was based on the estimation formula for comparing the means of two samples: n_1_-n_2_ = $\frac{\left(u_{\alpha }+u_{\beta }\right)\;2\times \left(\sigma +\sigma_{21}^{2}\right)}{\delta^{2}}$ . When α = 0.05 and β = 0.2, a two-sided test was used. By referring to the table, u_α_ = 1.96 and u_β_ = 0.84 were obtained. With reference to the previous literature (Shen et al., 2023), based on the results of traditional Chinese medicine (TCM) symptom scores in it, σ_1_ was taken as 2.79, σ_2_ as 3.30, and δ as 1.50. Substituting these values into the formula, n = 65.07 was calculated. Considering a 5% dropout rate, it was determined that 69 cases should be included in each group, with a total sample size of 138 cases.

### Randomization method and blinding

The randomization scheme required for the study was generated using the PROC PLAN procedure of SAS 9.4 statistical analysis software. Subjects were randomly allocated to the control group and the combined group in a 1:1 ratio using block randomization, with 69 cases in each group. The researchers responsible for random allocation wrote the group assignments on cards and placed them in sealed, opaque envelopes. Researchers not involved in treatment or efficacy evaluation were responsible for enrolling patients and allocating them to the designated groups. The efficacy evaluators and data statistical analysts were blinded to the group assignments, meaning that blinding was implemented for the outcome evaluators and statisticians.

### Treatment methods

After admission, both groups of patients were given relevant basic therapeutic interventions, including expectorant therapy (nebulized inhalation of glucocorticoids, administration of bronchodilators, back tapping or back oscillation with an oscillator, and suctioning with a sputum aspirator), nutritional support (early enteral or parenteral nutritional support provided according to the patients’ specific conditions), anti-inflammatory treatment (administration of glucocorticoids based on the patients’ specific conditions), anti-infection therapy (empirical anti-infection treatment with broad-spectrum antibiotics), and respiratory support (use of non-invasive or innovative ventilators according to the patients’ conditions to maintain their peripheral oxygen saturation above 90%). On this basis, the control group received ambroxol hydrochloride Injection (Jiangsu Guodan Biopharmaceutical Co., Ltd., H20203179, 2 mL: 15 mg). A dose of 30 mg was mixed with 250 mL saline and administered via intravenous drip twice daily. While the combined group received modified Shenzhe Zhenqi Decoction in addition to ambroxol hydrochloride, with the same dosing regimen for ambroxol hydrochloride as in the control group. The herbal formula of Shenzhe Zhenqi Decoction included: ginseng 9 g, hematite 30 g, dragon bone 30 g, oyster shell 30 g, gordon euryale seed 30 g, cornus 10 g, rhizoma dioscoreae 15 g, perilla fruit 10 g, rhizoma pinelliae praeparatum 15 g, aged tangerine peel 10 g, white peony root 10 g, poria 15 g, black sesame 10 g, and arborvitae seed 10 g. Preparation method and taking requirements of the TCM decoction: These Chinese medicine were purchased by The First Affiliated Hospital of Xi’an Jiaotong University and made into decoction by the pharmacy department, each dose of Chinese medicine was boiled into 2 sachets, 150 mL per sachet, which was taken half an hour apart from the western medicines after meals, 2 times a day, 1 time 1 sachet. Both groups continued treatment for 2 weeks.

### Observational indicators

(1) TCM syndrome score: mainly comparing the four main symptoms: fever, cough, sputum and dyspnoea. Scoring principle: The assessment was conducted before and after treatment with reference to the *S. for Diagnosis and Therapeutic Effect of Traditional Chinese Medicine Diseases and Syndromes (ZY/T001.1-94).* Each principal symptom was defined as 0, 2, 4 and 6 points from the degree of symptomatic absence, mildness, moderateness and severity.
(2) Arterial blood gas indices: The three main items of oxygen saturation (SaO_2_), partial pressure of oxygen (PaO_2_) and partial pressure of carbon dioxide (PaCO_2_) were compared. Detection method: 4 mL of fasting radial artery blood was extracted from the two groups of patients before and after treatment, and was measured using an arterial blood gas analyser (GEM3500, Werfen Inc., United States), and the data were recorded and stored in a timely manner.
(3) Lung function: The three main items were compared: forced expiratory volume in one second (FEV1), forced vital capacity (FVC) and peak expiratory flow (PEF). Measurement method: The lung function was measured by a spirometer (MasterScreen Pneumo, Jäger, Germany) before and after the treatment, and the data were recorded and stored in a timely manner.
(4) Inflammatory factors: The three main items of interleukin-6 (IL-6), tumour necrosis factor-α (TNF-α) and C-reactive protein (CRP) were compared. Detection method: 5 mL of fasting venous blood was extracted from two groups of patients before and after treatment, and serum was separated and cryopreserved. Serum IL-6 and TNF-α levels were determined by enzyme-linked immunosorbent assay, and CRP levels were determined by immunoturbidimetric assay; the equipment and supporting reagents used were provided by Merck Company of the U.S.A., and the data were timely recorded and preserved.
(5) Adverse reactions and liver and kidney function indicators: Adverse reactions mainly included dizziness and headache, rash and itching, nausea and vomiting, abdominal pain and diarrhoea. These were closely observed and collected during the treatment period, and statistics were compiled after 2 weeks of treatment. Before and after treatment, liver and kidney function indicators of patients in both groups were detected using an automatic biochemical analyzer (Beckman, model: AU680, United States), including alanine aminotransferase (ALT), aspartate aminotransferase (AST), total bilirubin (TB), serum creatinine (Scr), and blood urea nitrogen (BUN).

### Clinical efficacy

Clinical efficacy was evaluated after 2 weeks based on the *S. for Diagnosis and Therapeutic Effect of Traditional Chinese Medicine Diseases and Syndromes (ZY/T001.1-94)*. Cured: All symptoms and signs such as fever, cough, expectoration, and dyspnea disappeared, with a reduction in TCM syndrome score of ≥70%, laboratory indicators within the normal range, and complete resolution of inflammatory exudates on imaging examination. Improved: Symptoms and signs such as fever, cough, expectoration, and dyspnea showed improvement, with a reduction in TCM syndrome score of ≥30%, improvement in laboratory indicators, and significant absorption of pulmonary inflammation on imaging examination. Uncured: Symptoms and signs such as fever, cough, expectoration, and dyspnea showed no significant improvement or worsened, with a reduction in TCM syndrome score of less than 30%, and no change or progression in laboratory indicators and imaging examination. Total efficacy rate = (cured + improved) cases/total cases × 100%.

### Statistics

Statistical analysis was conducted employing SPSS 26.0. Qualitative data were expressed as [n (%)], with χ^2^ testing. Quantitative data with normal distribution were represented as $\bar{x}$ ± s, and analyzed with independent or paired t-tests. Skewed quantitative data were presented as M (*P25, P75*), and analyzed adopting the *Mann-Whitney U* test. Differences were considered statistically significant when *P* < 0.05.

## Results

### Clinical efficacy

The total effective rate after treatment in the combined group and control group was 98.55% and 89.86%, with significant differences between the groups (*P* < 0.05) (Table 2).

**TABLE 2:** Comparison of clinical efficacy between the two groups [n (%) ].

Efficacy	Combined group (n = 69)	Control group (n = 69)	*χ* ^2^	*P*
Cured	55 (79.71)	44 (63.77)	—	—
Improved	13 (18.84)	18 (26.09)	—	—
Uncured	1 (1.45)	7 (10.14)	—	—
Total	68 (98.55)	62 (89.86)	4.777	0.029

### TCM syndrome scores

No significant differences in the scores of fever, cough, sputum and dyspnoea between the two groups before treatment were found (*P* > 0.05); scores of fever, cough, expectoration and dyspnoea of the two groups were reduced after treatment, and those of the combined group were lower than those of the control group (*P* < 0.05) (Table 3).

**TABLE 3:** Comparison of TCM syndrome scores between the two groups (points).

Time	Combined group (n = 69)	Control group (n = 69)	*Z*	*P*
Before treatment				
Fever	4.00 (4.00, 4.00)	4.00 (4.00, 4.00)	−0.154	0.878
Cough	6.00 (4.00, 6.00)	4.00 (4.00, 6.00)	−0.387	0.699
Expectoration	6.00 (4.00, 6.00)	6.00 (4.00, 6.00)	−0.510	0.610
Dyspnoea	6.00 (4.00, 6.00)	6.00 (4.00, 6.00)	0.350	0.726
After treatment				
Fever	0.00 (0.00, 0.00)^a^	0.00 (0.00, 0.00)^a^	−2.178	0.029
Cough	0.00 (0.00, 0.00)^a^	0.00 (0.00, 2.00)^a^	−2.251	0.024
expectoration	0.00 (0.00, 0.00)^a^	0.00 (0.00, 2.00)^a^	−2.502	0.012
Dyspnoea	0.00 (0.00, 2.00)^a^	0.00 (0.00, 2.00)^a^	−2.045	0.041

Note: ^a^ *P* < 0.05 vs. the same group before treatment.

### Arterial blood gas indices

There were no significant differences in SaO_2_, PaO_2_ and PaCO_2_ between the two groups (*P* > 0.05); SaO_2_ and PaO_2_ increased and PaCO_2_ decreased after treatment in the two groups, and all of them were improved in the combined group compared with in the control group (*P* < 0.05) (Table 4).

**TABLE 4:** Comparison of arterial blood gas indices between the two groups.

Time	Combined group (n = 69)	Control group (n = 69)	*Z*	*P*
Before treatment				
SaO_2_ (%)	74.00 (71.00, 78.50)	73.00 (68.00, 78.00)	−1.223	0.221
PaO_2_ (mmHg)	52.00 (49.00, 56.50)	52.00 (48.00, 56.00)	−0.587	0.557
PaCO_2_ (mmHg)	67.00 (63.50, 71.50)	69.00 (65.50, 72.00)	−1.586	0.113
After treatment				
SaO_2_ (%)	95.00 (94.00, 97.00)^a^	94.00 (93.00, 95.00)^a^	−3.886	<0.001
PaO_2_ (mmHg)	83.00 (78.00, 87.50)^a^	78.00 (74.00, 82.00)^a^	−4.484	<0.001
PaCO_2_ (mmHg)	43.00 (38.00, 49.00)^a^	51.00 (47.00, 56.00)^a^	−6.241	<0.001

Note: ^a^ *P* < 0.05 vs. the same group before treatment.

### Lung function

No significant differences in FEV1, FVC, and PEF between the two groups before treatment were observed (*P* > 0.05); FEV1, FVC, and PEF increased in both groups after treatment, and they were all higher in the combined group than in the control group (*P* < 0.05) (Table 5).

**TABLE 5:** Comparison of lung function between two groups.

Time	Combined group (n = 69)	Control group (n = 69)	*Z/t*	*P*
Before treatment				
FEV1 (L)	1.19 (0.95, 1.56)	1.23 (0.94, 1.56)	−0.166	0.868
FVC (L)	1.82 ± 0.32	1.84 ± 0.28	−0.387	0.700
PEF (L/s)	1.65 ± 0.29	1.67 ± 0.27	−0.419	0.676
After treatment				
FEV1 (L)	2.21 (1.97, 2.48)^a^	1.75 (1.47, 2.05)^a^	−6.703	<0.001
FVC (L)	2.92 ± 0.38^a^	2.53 ± 0.45^a^	5.501	<0.001
PEF (L/s)	2.89 ± 0.20^a^	2.64 ± 0.29^a^	5.817	<0.001

Note: ^a^ *P* < 0.05 vs. the same group before treatment.

### Inflammatory factors

There were no marked differences in serum IL-6, TNF-α and CRP levels between the two groups (*P* > 0.05); serum IL-6, TNF-α and CRP levels were reduced in both groups after treatment, and they were lower in the combined group than in the control group (*P* < 0.05) (Table 6).

**TABLE 6:** Comparison of inflammatory factors between the two groups.

Time	Combined group (n = 69)	Control group (n = 69)	*t/Z*	*P*
Before treatment				
IL-6 (ng/L)	96.42 ± 6.90	97.90 ± 7.65	−1.194	0.235
TNF-α (ng/L)	70.34 ± 4.50	69.15 ± 4.75	1.510	0.133
CRP (mg/L)	98.60 (91.74, 106.55)	96.87 (89.61, 105.28)	−1.079	0.280
After treatment				
IL-6 (ng/L)	19.58 ± 5.94^a^	23.33 ± 6.84^a^	−3.440	0.001
TNF-α (ng/L)	9.47 ± 4.19^a^	13.45 ± 4.34^a^	−5.478	<0.001
CRP (mg/L)	5.87 (2.61, 9.75)^a^	9.13 (4.85, 14.10)^a^	−3.507	<0.001

Note: ^a^ *P* < 0.05 vs. the same group before treatment.

### Adverse reactions and liver and kidney function indicators

The total incidence of adverse reactions during treatment in the combined group and the control group was 4.35% and 2.90%, with no significant differences between the groups (*P* > 0.05) (Table 7). There was no statistically significant difference in liver function indicators (ALT, AST, and TB) and kidney function indicators (Scr, BUN) between the two groups before treatment (*P* > 0.05). There were no significant differences in ALT, AST, TB, Scr, and BUN between the two groups after treatment compared with before treatment (*P* > 0.05), and there was no statistically significant difference between the two groups (*P* > 0.05) (Table 8).

**TABLE 7:** Comparison of adverse reactions between the two groups [n (%) ].

Adverse reaction	Combined group (n = 69)	Control group (n = 69)	*χ* ^2^	*P*
Dizziness and headache	0 (0.00)	1 (1.45)	—	—
Rash and itching	1 (1.45)	0 (0.00)	—	—
Nausea and vomiting	1 (1.45)	0 (0.00)	—	—
Abdominal pain and diarrhoea	1 (1.45)	1 (1.45)	—	—
Total	3 (4.35)	2 (2.90)	0.208	0.649

**TABLE 8:** Comparison of liver and kidney function between the two groups.

Time	Combined group (n = 69)	Control group (n = 69)	t	*P*
Before treatment				
ALT (U/L)	33.39 ± 11.50	35.25 ± 16.36	0.773	0.441
AST (U/L)	27.30 ± 8.25	28.36 ± 10.54	0.658	0.512
TB (μmol/L)	11.92 ± 3.71	12.33 ± 3.42	0.675	0.501
Scr (μmol/L)	60.50 ± 18.52	61.23 ± 18.33	0.233	0.816
BUN (mmol/L)	5.54 ± 1.63	5.12 ± 1.56	1.546	0.125
After treatment				
ALT (U/L)	34.65 ± 14.26	36.28 ± 17.52	0.600	0.550
AST (U/L)	29.64 ± 10.06	30.25 ± 11.10	0.338	0.736
TB (μmol/L)	10.08 ± 2.95	11.77 ± 2.38	1.509	0.134
Scr (μmol/L)	62.72 ± 19.64	63.39 ± 16.84	0.215	0.830
BUN (mmol/L)	5.47 ± 1.57	5.26 ± 1.62	0.770	0.443

## Discussion

Pneumonia continues to be one of the leading causes of mortality (Hespanhol and Barbara, 2020). Despite being a frequently encountered issue in clinical practice, pneumonia poses significant challenges in accurate diagnosis and treatment due to its varying presentations, causative organisms, and severity (Lanks et al., 2019). Some studies have discussed the treatment of pneumonia and the efficacy of different drugs for different types of pneumonia, such as the impacts of combining N-Acetylcysteine with ambroxol hydrochloride on clinical symptoms (Xue et al., 2024). Our study explored the synergistic effects of modified Shenzhe Zhenqi Decoction and ambroxol hydrochloride in the treatment of severe pneumonia. Our findings suggested that a combination of modified Shenzhe Zhenqi Decoction and ambroxol hydrochloride may offer enhanced therapeutic benefits by addressing different aspects of the disease simultaneously, which may lead to better patient outcomes compared to monotherapy.

Specifically, the combined treatment group achieved better therapeutic efficacy in improving TCM syndrome scores (relief of symptoms such as fever, cough, sputum production, and dyspnea). A previous study has also suggested that in the recovery of children with severe pneumonia, the time required for fever clearance, cough disappearance, improvement in lung function, chest shadow absorption, and hospital discharge are shortened with the use of ambroxol hydrochloride (Yu et al., 2021). Although there are no studies demonstrating the efficacy of Shenzhe Zhenqi Decoction in the treatment of pneumonia, there are a number of studies on the treatment of pneumonia with TCM decoction. Huashi Baidu decoction (Q-14) is a well-known TCM decoction with evidence of notable efficacy in the treatment of pneumonia, improvement of influenza-like symptoms, and potent antiviral and anti-inflammatory effects (Xu et al., 2023). Two of the herbs contained in Q-14 (pinelliae praeparatum and poria) are also found in Shenzhe Zhenqi Decoction, which indirectly suggests that Shenzhe Zhenqi Decoction may also have a similar efficacy in improving the symptoms of pneumonia.

In addition, compared with the control group, the combined group demonstrated more significant improvements in arterial blood gas indices (SaO_2_, PaO_2_, and PaCO_2_), along with higher values of FEV1, FVC, and PEF. This indicates that the treatment of severe pneumonia with modified Shenzhe Zhenqi Decoction combined with ambroxol hydrochloride can improve arterial blood gas indices and lung function. Xiaoqinglong decoction has been shown to have a significant effect on improving arterial blood gas (Su et al., 2022), and the fact that this decoction also contains pinelliae praeparatum suggests that Shenzhe Zhenqi Decoction may also have such an effect. Furthermore, a prior study has demonstrated that ambroxol hydrochloride is capable of significantly enhancing the lung function of elderly patients, exhibiting notable clinical efficacy and a high level of drug safety (Li, 2021). Additionally, Maimendong decoction, a traditional Chinese herbal formula containing ginseng, pinelliae praeparatum, and cornus, has demonstrated efficacy in enhancing pulmonary function (Lao et al., 2024), all of which are found in Shenzhe Zhenqi Decoction, suggesting that Shenzhe Zhenqi Decoction also has this effect. Meanwhile, inflammatory factors, including IL-6, TNF-α, and CRP, were reduced, and they were lower in the combined group than in the control group, reflecting a robust anti-inflammatory effect. This fact has also been confirmed in a previous study which showed that ambroxol alleviates lung inflammation and reduces the secretion of pro-inflammatory cytokines, including IL-6, TNF-α, and TGF-β (Khoury et al., 2020). Importantly, Shenzhe Zhenqi Decoction is composed of multiple Chinese herbal medicines, each with distinct pharmacological effects that may collectively exert anti-inflammatory and immunomodulatory effects. Ginseng contains various saponin components, which have notable anti-inflammatory and immunomodulatory actions. It can inhibit the release of inflammatory mediators and regulate the function of immune cells, thereby alleviating inflammatory responses (Zheng et al., 2024). Meanwhile, hematite has the effects of calming the adverse rising qi, resolving phlegm, and relieving asthma. It may indirectly enhance anti-inflammatory effects by regulating the overall state of the body (Lin, 2022). White peony root contains components such as paeoniflorin, which possess anti-inflammatory, analgesic, and immunomodulatory properties. It can inhibit the production of inflammatory mediators, alleviate inflammatory responses, and may exert immunomodulatory effects by regulating the function of immune cells (Liu et al., 2025). Other ingredients, such as gordon euryale seed, rhizoma dioscoreae, and cornus, may also have certain anti-inflammatory and immunomodulatory effects, but the specific mechanisms remain to be further studied. Future in-depth experimental research and clinical validation can be conducted. Methods such as *in vitro* experiments, animal experiments, and clinical trials can be employed to separately investigate the effects of each ingredient on inflammatory responses and their interactions, thereby revealing the molecular mechanisms underlying the anti-inflammatory effects of Shenzhe Zhenqi Decoction.

In terms of overall clinical efficacy, the total effective rate in the combined treatment group reached 98.55%, significantly higher than the 89.86% observed in the control group. The mechanism underlying these outcomes is likely attributable to the distinct therapeutic actions of each component. Modified Shenzhe Zhenqi Decoction is known for its heat-clearing, detoxifying, and Qi-tonifying properties. Its ingredients have been proven in many studies to be able to enhance anti-inflammatory responses, arterial blood gas indices and lung functions, thereby improving symptoms in patients with severe pneumonia. Meanwhile, ambroxol hydrochloride boosts the production of lung fluid and alveoli. Furthermore, it has multiple roles, including helping to break down thick mucus, making it less sticky, encouraging movement in the bronchial cilia, and assisting in the removal of thick phlegm (Liu et al., 2024). Together, these therapies exert synergistic effects in reducing pulmonary inflammation, enhancing ventilation, and improving blood oxygenation. These findings are consistent with prior research supporting the efficacy of integrative treatments for severe pneumonia, further confirming the therapeutic potential of this combination.

In conclusion, the results demonstrate that the combined treatment group, which received modified Shenzhe Zhenqi Decoction alongside ambroxol hydrochloride, showed notable improvements across clinical symptoms, arterial blood gas indices, lung function, inflammatory factors, and overall therapeutic efficacy, compared to the group treated with ambroxol hydrochloride alone. This outcome highlights the efficacy of the integrative approach. It is worth noting that this study also has some limitations. For instance, the limited sample size of this study may affect the generalizability of the results. Additionally, severe pneumonia has diverse etiologies, including infections by various pathogens such as bacteria, viruses, fungi, and *mycoplasma*, as well as non-infectious factors. This study did not subgroup patients based on different etiologies or pathogens, which may prevent an accurate and comprehensive assessment of the differences in the therapeutic efficacy of modified Shenzhe Zhenqi Decoction across varying etiological subtypes of severe pneumonia. In the future, multi-center, large-sample clinical studies should be conducted to more comprehensively evaluate the efficacy and safety of modified Shenzhe Zhenqi Decoction in treating severe pneumonia. Patients should be subgrouped based on different etiologies and pathogens to deeply explore the differences in the therapeutic efficacy of modified Shenzhe Zhenqi Decoction for severe pneumonia caused by different etiologies and pathogens. Meanwhile, further in-depth research on the mechanism of action of modified Shenzhe Zhenqi Decoction should be carried out to clarify the specific targets and signaling pathways of its anti-inflammatory, immunomodulatory, and other effects. This will also help provide a more scientific theoretical basis for the clinical application of modified Shenzhe Zhenqi Decoction.

## Data Availability

The original contributions presented in the study are included in the article/supplementary material, further inquiries can be directed to the corresponding author.
